# Comparative Genomic and Functional Evaluations of *Bacillus subtilis* Newly Isolated from Korean Traditional Fermented Foods

**DOI:** 10.3390/foods9121805

**Published:** 2020-12-04

**Authors:** Hye Jin Choi, Donghyun Shin, Minhye Shin, Bohyun Yun, Minkyoung Kang, Hee-Jong Yang, Do-Youn Jeong, Younghoon Kim, Sangnam Oh

**Affiliations:** 1 Department of Agricultural Biotechnology and Research Institute of Agriculture and Life Science, Seoul National University, Seoul 08826, Korea; zni901@snu.ac.kr (H.J.C.); mhshin1984@snu.ac.kr (M.S.); 2 Department of Agricultural Convergence Technology, Jeonbuk National University, Jeonju 54896, Korea; sdh1214@gmail.com; 3 Department of Animal Science and Institute of Milk Genomics, Jeonbuk National University, Jeonju 54896, Korea; boding3@nate.com; 4 Department of Functional Food and Biotechnology, Jeonju University, Jeonju 55069, Korea; mink118283@gmail.com; 5 Microbial Institute for Fermentation Industry, Sunchang, Jeonbuk 56048, Korea; godfiltss@naver.com (H.-J.Y.); jdy2534@korea.kr (D.-Y.J.)

**Keywords:** probiotics, anti-aging, Korean traditional fermented foods, *Caenorhabditis elegans*

## Abstract

Many fermented foods are known to have beneficial effects on human and animal health, offering anti-aging and immunomodulatory benefits to host. Microorganisms contained in the fermented foods are known to provide metabolic products possibly improving host health. However, despite of a number of studies on the functional effects of the fermented foods, isolation and identification of the effective bacterial strains in the products are still in progress. The objective of this study was to isolate candidate functional strains in various Korean traditional fermented foods, including ganjang, gochujang, doenjang, and jeotgal, and evaluate their beneficial effects on the host, using *Caenorhabditis elegans* as a surrogate animal model. Among the 30 strains isolated, five *Bacillus* spp. were selected that increased the expression level of *pmk-1*, an innate immune gene of *C. elegans*. These strains extended the nematode lifespan and showed intestinal adhesion to the host. Based on the bioinformatic analyses of whole genome sequences and pangenomes, the five strains of *Bacillus subtilis* were genetically different from the strains found in East Asian countries and previously reported strains isolated from Korean fermented foods. Our findings suggest that the newly isolated *B. subtilis* strains can be a good candidate for probiotic with further in-depth investigation on health benefits and safety.

## 1. Introduction

With increased consumer interest in functional foods, the food industry has experienced innovative and economic expansion [1]. A number of studies have reported that functional foods provide benefits to host health functions and anti-aging by enhancing host immunity [2]. These health-promoting functions can be further increased by microbes that propagate during the process of food fermentation [3]. The microorganisms produce metabolic products such as proteases, antibiotic metabolites, and signaling molecules to the host cells, regulating the host immune responses and metabolism [4,5,6].

The WHO has defined probiotics as “live microorganisms that, when administered in adequate amounts, confer a health benefit on the host” [7]. Probiotics are known to improve gut microbial balance, strengthen the immune system, and reduce specific cancer risk and have anti-aging effects [8,9,10]. In particular, a number of probiotics are involved in the fermentation of Korean traditional fermented foods such as ganjang (fermented soy sauce), gochujang (fermented red chilli paste), doenjang (fermented soybean paste), and kimchi (fermented pickled vegetables), increasing the nutritional and functional potential of the foods [11]. Representatively, microorganisms involved in the fermentation process of Korean traditional fermented foods include *Bacillus* spp., *Leuconostoc* spp., *Weissella* spp., *Aspergillus* spp., *Mucor* spp., and *Lactobacillus* spp. [12]. These microorganisms derived from the fermented foods may provide a positive effect on host intestinal microorganisms, reducing pathogenic bacterial growth and stimulating the immune responses [13]. Especially, *Bacillus* species have been proven to be commonly used probiotic strains with health benefits and are symbiotic microorganisms that also exist in the human gastrointestinal tract [14]. They are gram-positive, either aerobic or anaerobic, and form endospores, giving them advantages of being stored at room temperature as a dry form over other non-spore-forming bacteria such as *Lactobacillus* species. They are also able to survive in the host gastrointestinal tract and reach the small intestine [15]. In particular, *B. subtilis* is a generally recognized as safe (GRAS) organism and has been investigated for animal probiotic development due to its high level of hydrolytic and fibrinolytic enzymes, secretion of antimicrobial compounds, and for producing spores cultured in aerobic conditions [16].

*Caenorhabditis elegans* is known to be suitable for experimental animal models because of its short life span and facile genetic manipulation [17]. It has been well established that *C. elegans* is a useful animal model for screening probiotic bacteria because its intestinal cell structure is similar to that of humans [18]. In this study, we isolated bacterial strains from Korean traditional fermented foods and identified *Bacillus* strains by 16S rRNA sequencing. *C. elegans* as a surrogate animal model was used to evaluate the effects of the isolated *Bacillus* spp. on the longevity and immunity enhancement of the host and intestinal adherence of strains. We also analyzed pangenomes based on whole-genome sequencing data of the isolated strains. Our findings suggest that the newly isolated *B. subtilis* strains can be used as promising anti-aging probiotic strains with the abilities to enhance immunity and prolong the lifespan of nematodes.

## 2. Materials and Methods

### 2.1. Bacterial Isolation and Culture

To isolate *Bacillus* spp., which can be used as probiotics, Korean traditional fermented foods such as Korean soy sauce (ganjang), Korean red pepper paste (gochujang), Korean soybean paste (doenjang), and Korean salted seafood (jeotgal) were collected from 5 different provinces in Korea, including Gyeonggi-do, Gangwon-do, Chungcheong-do, Jeolla-do, and Gyeongsang-do (fermentation period: August 2017 to November 2019). One gram of each sample collected was mixed with 9 mL of sterile water and serially diluted. The diluted samples were spread on Luria-Bertani (LB) agar (Difco, Sparks, MD, USA), and colonies were selected after incubation at 30 °C for 18 h. It is noted that our procedure was not selective for spore forming cells and that we selected colonies using the morphological differences such as smooth, humid, and mucoid phenotypes on LB agar plate [19]. Further 16S rRNA sequencing was conducted for identification of the strains. *Lactocaseibacillus rhamnosus* GG (LGG), used for a control in the nematode associated analyses, was grown in MRS medium at 37 °C.

### 2.2. Acid Tolerance and Bile Tolerance

To evaluate the acid resistance of selected *Bacillus* spp., bacterial isolates were inoculated in LB broth (5 mL) at pH 2.0 adjusted with 5 N HCl (Sigma-Aldrich, St. Louis, MO, USA) and incubated at 30 °C for 1 h with agitation. After serial dilution, the culture medium was plated on LB agar medium and incubated in a 30 °C incubator, and the acid resistance was analyzed by measuring the number of viable cells. The control group was cultured in LB medium without adjusting the pH. In parallel, for bile resistance, isolates were inoculated in LB broth containing 0.3% Oxgall (Neogen, MI, USA) and cultivated for 30 h in a shaking incubator at 250 rpm. Oxgall is manufactured from fresh bile by rapid evaporation of the water content and composed of fatty acids, bile acids, inorganic salts, sulfates, bile pigments, cholesterol, mucin, lecithin, glycuronicaids, porphyrins, and urea. After serial dilution of the culture medium, the cells were plated on LB agar medium and cultured in a 30 °C incubator, and the resistance characteristics were analyzed by measuring the number of viable cells [20].

### 2.3. Antimicrobial Activity

Indicator strains (*Bacillus cereus*, *Staphylococcus aureus*, *Listeria monocytogenes* and *Enterococcus faecalis*) for antimicrobial activity were obtained from the Korean Culture Center of Microorganisms (KCCM, Seoul, Korea) and the Korean Collection for Type Cultures (KCTC, Daejeon, Korea). Accession numbers are KCTC3624 and KCCM40935 for *B. cereus*, KCCM11335 for *S. aureus*, KCCM43155 for *L. monocytogenes*, and KCCM11814 for *E. faecalis*. According to the agar diffusion method [21], each pathogenic strain was cultured to an OD (optical density) of 0.4 to 0.6 at 600 nm in an appropriate broth medium including LB or Tryptic Soy Broth (TSB), mixed with 1% in 0.8% of molten agar medium, and subsequently solidified for incubation of the test strains. The *Bacillus* isolates were inoculated in 5 mL of LB broth and incubated in a 30 °C with 150 rpm shaking in an incubator for 24 h. The culture supernatant was prepared by centrifugation at 13,000 rpm and filtration using a 0.45-µm syringe filter. One hundred microliters of the solution were added into an 8-mm hole in the agar medium containing each pathogenic strain and incubated for 24 h at 30 or 37 °C depending on the pathogenic strains. The antimicrobial activity of the selected strains was evaluated by the diameter of the zone of inhibition.

### 2.4. C. elegans Culture Conditions

The *C. elegans* strains used in this study were CF512 *fer-15*(b26)II;*fem-1*(hc17) IV (*fer-15*;*fem-1* worms) and AY102 (P-*vha-6*::*pmk-1*::GFP +*rol-6*(su1006);*pmk-1*::GFP). For survival experiments, the *fer-15*;*fem-1* mutants were used because they are unable to produce progeny at 25 °C without alteration in the *C. elegans* phenotype. In addition, we employed the AY102 mutant strain harboring the GFP expression system for the activation of *pmk-1* (the worm homologue of p38 MAPK), which is a key player in the immune response and aging in *C. elegans* [17]. Worms were maintained on nematode growth medium (NGM) plates seeded with *E. coli* OP50 and maintained at 25 °C as described previously [22].

### 2.5. C. elegans Pmk-1-Mediated Screening for Anti-Aging Activity

The anti-aging activity assay using AY102 transgenic worms at the L4 stage was performed by modifying the previously described method [22]. Briefly, the worms were exposed to NGM plates treated with probiotic candidate strains (10^7^ colony forming units per plate) for 24 h. After exposing *C. elegans* to preconditioning plates of *Bacillus* isolates for 24 h, 10 worms were picked randomly and placed on Brain Heart Infusion (BHI) plates containing 100 μg/mL kanamycin (Sigma-Aldrich) and 100 μg/mL streptomycin (Sigma-Aldrich). Three to five worms were transferred to a round-bottom 96-well plate containing 20 μL of M9 buffer and anesthetized by treatment with 20 μL of 30 mM NaN_3_ (Sigma-Aldrich). Fluorescence microscopy (IX53, Olympus, Tokyo, Japan) was used to determine the expression rate of GFP (green fluorescent protein) in *C. elegans*. Images were analyzed using ImageJ (https://imagej.nih.gov/ij).

### 2.6. C. elegans Lifespan Analysis

The lifespan of *C. elegans* was measured as in previous studies to confirm that candidate organisms can prolong the survival of *C. elegans* [23]. The worms at the L4 stage were exposed to bacteria-treated NGM plates (5 × 10^6^ colony forming units per plate), and the number of living worms was recorded daily. To accurately count, worms were moved to new plate with bacteria every 3 days. Worms were considered dead when they did not respond to a careful touch, and all *C. elegans* were incubated at 25 °C.

### 2.7. Measurement of Intestinal Colonization of C. elegans

To measure the colonization of *C. elegans*, the numbers of bacterial cells in worm intestines were measured. The amounts of bacteria adhered to the intestine of the worm were measured according to the method described above [18]. Briefly, after exposing *C. elegans* (at the L4 stage) to each isolated bacterium (*E. coli* OP50 as a non-colonizing negative control, LGG as a colonizing positive control, or each isolated *Bacillus* strain) for 24 h, 10 worms were picked randomly, washed twice in M9 buffer, and placed on BHI plates containing 100 μg/mL kanamycin (Sigma-Aldrich) and 100 μg/mL streptomycin (Sigma-Aldrich). To remove the bacteria attached to the surface of the worm, the worms were washed with 5 μL of gentamicin solution (25 μg/mL) for 5 min. The worms were washed 5 times with M9 buffer, treated with 1% Triton X-100 dissolved in M9 buffer, and physically disrupted using a pestle motor. The diluted worm lysates were plated on acidified MRS agar plates (pH 5.0) for LGG at 37 °C for 24 h or on LB agar plates for *E. coli* OP50 and *Bacillus* strains at 30 °C for 24 h.

### 2.8. Whole-Genome Sequencing and Pangenome Analysis

Whole-genome shotgun sequencing of five *B. subtilis* bacteria, SRCM103517, SRCM103571, SRCM103576, SRCM103689, and SRCM104011, was carried out using PacBio SMRT sequencing technology. A sequence of nucleotides was generated by DNA polymerase incorporation with a circular single molecule real-time (SMRT) sequencing system. Polymerase reads were trimmed to include only the high-quality regional sequences from adapters and further to include sequences from multiple passes around a circular template. Each polymerase read was partitioned to form one or more subreads, which contained sequences from a single pass of a polymerase on a single strand of an insert within an SMRTbell ™ template (PacBio, Menlo Park, CA, USA). The sequencing library was prepared by random fragmentation of the DNA or the cDNA sample, followed by 5′ and 3′ adapter ligation. This library was loaded into a flow cell where the fragments are captured on a lawn of surface-bound oligos complementary to the library adapters. As all four reversible, terminator-bound dNTPs are present during each sequencing cycle, the natural competition minimizes incorporation bias and significantly reduces raw error rates compared with other technologies. Then, the sequencing data were converted into raw data for analysis. Contigs were constructed by de novo assembly using the Hierarchical Genome Assembly Process(HGAP) Assembly (v. 3.0, PacBio, Menlo Park, CA, USA), which was initially preassembling seed reads, generating a consensus sequence of the mapped reads and correcting and filtering the reads. The genomic sequences were deposited in the NCBI sequence Read Archive under project accession number PRJNA515340 (for SRCM103517), PRJNA515369 (for SRCM103571), PRJNA516537 (for SRCM103576), PRJNA516532 (for SRCM103689), and PRJNA515144 (for SRCM104011).

For pangenome analysis, the genome sequences of 24 strains isolated from food in Korea and 32 strains from various hosts in China and Japan were acquired from GenBank to compare different host-derived strains. All 61 genome sequences, including 5 sequenced in this study, were annotated by Prokka (v 1.14.5, Victorian Bioinformatics Consortium, https://vicbioinformatics.com/software.prokka.shtml), and the protein coding sequences were predicted by the EggNOG-mapper (v 2.0.1, http://eggnog-mapper.embl.de) and functionally categorized based on the COG database. To evaluate genetic relatedness, the average nucleotide identity (ANI) was calculated based on the JSpecies web server (http://jspecies.ribohost.com/jspeciesws).

The nucleotide sequences of 61 *B. subtilis* strains were annotated by Prokka to obtain GFF format files, which were used to calculate the core and pangenomes. The core and pangenomes were calculated using Roary (v 3.13.0, https://sanger-pathogens.github.io/Roary), a rapid standalone pangenomic pipeline. A pair of genes was defined as belonging to the same gene family when the identity value of their amino acid sequences was >95%. In addition, the phylogenetic tree was constructed based on the core genes using Randomized Accelerated Maximum Likelihood (RAxML) (v 8.2.12, https://github.com/stamatak/standard-RAxML) and was drawn by MEGA-X (v 10.0.5., https://www.megasoftware.net) from the newick file.

### 2.9. Statistical Analysis

The survival rate of worms was assessed using the Kaplan–Meier method, and the significance of differences was determined using a log-rank test between survival curves (STATA6, STATA, College Station, TX, USA). We conducted Student’s *t*-tests to determine significant differences in colony-forming unit (CFU) calculations to determine the presence of bacteria. All data represent three independent results. A *p* value of 0.05 in all replicates was considered to reflect a significant difference when compared to the control.

## 3. Results and Discussion

### 3.1. Functional Analysis of Potential Probiotics

One of the beneficial effect of probiotics on host as a living organism is to improve the intestinal microbial balance, implementing that they must be able to survive at the low pH and with bile salts of the gastrointestinal environment [24]. To conduct primary screening of potential probiotic strains in vitro, we evaluated the acid and bile tolerances of the bacterial strains isolated from Korean traditional foods (described in the Section 2). A total of 30 candidate *Bacillus* strains, identified by 16S rRNA sequencing, were tested, and subsequently, 16 strains showed survival rates of 50% or higher at pH 2, and 28 strains showed survival rates of 80% or higher at 0.3% bile acid (Supplementary Figure S1). *B. subtilis* SRCM103612, the highest acid-resistant strain, showed 75.91% survival at pH 2, while *B. velezensis* SRCM103691, the highest bile-resistant strain, had 99.09% survival at 0.3% bile acid.

We next assessed antimicrobial activity against pathogenic bacteria such as *B. cereus*, *S. aureus*, *L. monocytogenes,* and *E. faecalis* to evaluate the antimicrobial activity of *Bacillus* spp. isolates from traditional Korean fermented foods. As a result of the antimicrobial activity test, 12 of the 30 candidate strains (SRCM103517, SRCM103571, SRCM103574, SRCM103581, SRCM103622, SRCM103623, SRCM103629, SRCM103837, SRCM103881, SRCM104005, SRCM104008, SRCM104011) had antimicrobial activity against all 5 pathogenic bacteria, and 17 strains (SRCM103551, SRCM103576, SRCM103583, SRCM103608, SRCM103612, SRCM103616, SRCM103639, SRCM103641, SRCM103689, SRCM103691, SRCM103696, SRCM103697, SRCM103773, SRCM103835, SRCM103844, SRCM103862, SRCM103923) except one strain (SRCM103788) had antimicrobial activity against one or more pathogens (Supplementary Table S1).

Korean traditional fermented foods are known to have functional properties such as fibrinolytic activity, antimicrobial activity, and hypocholesterolemic effects [25]. In particular, *Bacillus* spp., generally present as 10^6^–10^7^ colony forming units (CFU) per ml in the products, is highly involved in the fermentation process of these fermented foods, affecting their quality as well as host effects such as cholesterol and fibrin decomposition activities [26,27]. For its use as a probiotic strain, the ingested bacteria must face the challenge of acidic environment and toxic compounds like bile during the passage through the gastrointestinal tract. *B. subtilis* is generally known as a neutrophile, but it also induces physiological systems that increase survival in extreme acidic conditions [28]. These systems include upregulation of dehydrogenases, decarboxylases, and SigX extracytoplasmic stress regulon. It is noteworthy to mention that *bacilli* tend to form spores at low pH [29]. The formation of spores would affect the bacterial ability for acid tolerance. Supplementary experiments to test the presence of spores at acidic condition will be required. In addition to the acid resistance, *bacilli* have a wide range of bile salt tolerance depending on the specific strains [30]. According to Wang et al., while *B. cereus* is sensitive to bile salts, *B. subtilis* and *B. amyloliquefaciens* perform well in tolerance to them [31]. In this study, we found that 16 and 28 species among 30 *Bacillus* isolates showed more than 50% acid resistance at pH 2.0 and more than 80% survival rate at 0.3% bile acid, respectively, indicating their potential uses as probiotics to survive against the host digestion process (Supplementary Figure S1).

*Bacillus*, which has a number of hydrolytic activities as well as produce a variety of antimicrobial peptides [32], has antimicrobial activity against pathogenic microorganisms such as *E. coli* O157 and *S. aureus*, and studies have shown that this antimicrobial activity can inhibit the growth of harmful microorganisms in food [33]. It is common that *Bacillus* strains possess an antimicrobial activity against a wide range of pathogenic bacteria including *B. cereus*, *S. aureus*, and *L. monocytogenes* by producing hydrolytic enzymes and antimicrobial peptides. Moreover, *B. subtilis* is able to produce a wide spectrum of bioactive antibiotic metabolites based on their biosynthetic pathways such as ribosomal peptides, volatile compounds, and polyketides [34]. Of the 30 bacteria isolated in this study, 29 bacterial strains, excluding one strain, had antibacterial activity against at least one representative pathogen, and 12 strains showed antibacterial activity against all 5 pathogens, which can function as potential probiotics (Supplementary Table S1). In order to support the results of antimicrobial activity in the selected *B. subtilis*, we analyzed presence of *sdpC* gene, encoding sporulation delaying protein C acting as an antimicrobial mediator, in their whole genome sequences, but could not find a general feature conserved for their antimicrobial activity. Further analysis on genetic differences among the strains will be investigated.

### 3.2. Screening of Probiotics That Have an Anti-Aging Effect on C. elegans

**Figure 1:**
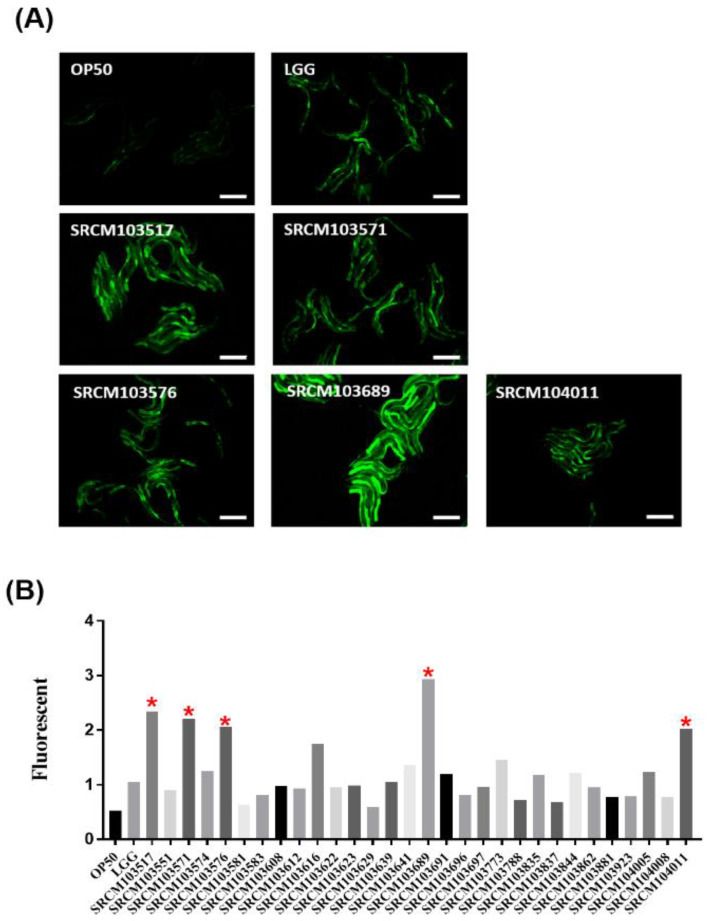
Induction of *pmk-1*::GFP exposed to candidate probiotics. (**A**) GFP visualization was assessed in the L4 stage of AY102 worms exposed to bacterial strains for 24 h. Animals integrated with the *pmk-1*::GFP transgene were visualized by fluorescence microscopy. The scale bar indicates 1 mm. (**B**) The degree of expression was measured using the ImageJ program and was then divided by the number of worms. *Bacillus subtilis* SRCM103689 showed the highest expression rate among the candidate strains. Red stars indicate five *Bacillus subtilis* strains showing high expression rate among the candidate strains.

The *C. elegans* model was used to select strains that could positively influence the longevity and immunity of the host [18]. Increasing innate immunity of nematode intestinal epithelial cells is beneficial for survival against exposure to pathogenic bacteria [35]. Innate immunity in *C. elegans* is regulated by the PMK-1 p38 mitogen-activated protein kinase (MAPK) pathway, which is essential for resistance against pathogenic bacteria and fungi [36]. To observe the stimulation of *pmk-1* in the nematode, the mutant animal AY102 *C. elegans* (P-*vha-6*::*pmk-1*::GFP +*rol-6*(su1006); *pmk-1*::GFP) was exposed to a bacteria-treated plate for 24 h [37]. Figure 1 shows the expression rate of stimulation of *pmk-1* via transgenic worms harboring the *pmk-1*::GFP reporter system with candidate bacteria. LGG was used as a positive probiotic control, which has been shown to have gastrointestinal and immune-enhancing functions [38,39,40]. Among 30 *Bacillus* spp., 14 strains were found to express higher levels of *pmk-1* than LGG, and the top 5 strains, *B. subtilis* SRCM103517, SRCM103571, SRCM103576, SRCM103689, and SRCM104011, were selected for further analysis (Figure 1).

The *pmk-1* pathway is known as a key pathway for the immune response of *C. elegans*, functioning to strengthen immunity of the nematode [41,42]. According to the previous studies, probiotics such as *lactobacilli* can increase nematode immunity and life span by increasing the expression of *pmk-1* and protect nematodes from pathogen infection [43,44]. The signaling pathway through PMK-1 is a key component of the *C. elegans* immune conditioning and promotion with probiotic strains [45]. In addition to *lactobacilli*, *B. subtilis* has been demonstrated to promote *C. elegans* innate immunity by producing a lipopeptide fengycin that mediates microbial antagonism especially against Gram-positive pathogens [43]. The bacteria isolated in this study, specifically the 5 selected bacteria, had higher expression levels of *pmk-1* than LGG, which could function in the host by stimulating innate immunity, here the *pmk-1* pathway in *C. elegans*. PMK-1 and insulin/insulin-like growth factor-1 (IIS) are well known pathways related to the immune response and longevity of *C. elegans* [46]. Besides PMK-1, the IIS pathway is the first pathway established in aging research and well known to regulate nematode growth, aging, and longevity [47]. In order to understand the mechanism of immunomodulatory activity in *C. elegans* with the specific 5 strains of *B. subtilis*, further transcriptional analyses will be required.

### 3.3. Bacillus Extends the Lifespan of C. elegans

**Figure 2:**
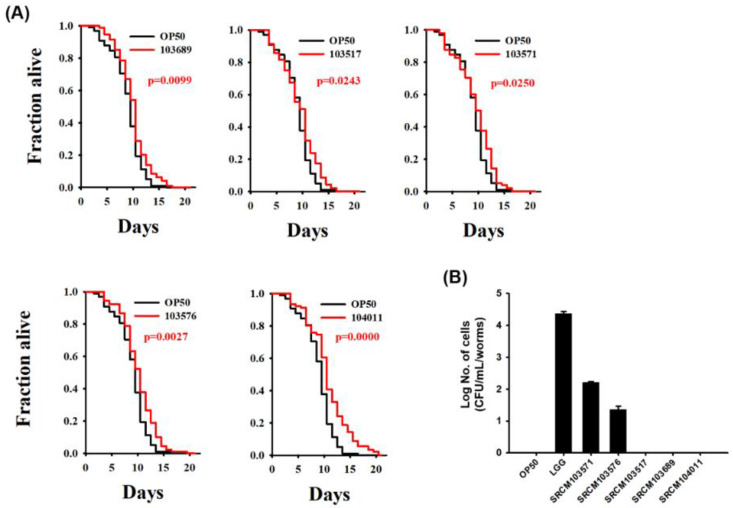
Anti-aging effects (**A**) and persistence (**B**) of selected *Bacillus subtilis* strains in *C. elegans*. (**A**) Survival statistics of *B. subtilis* were compared with the worms exposed to *E. coli* OP50, with *p* = 0.0243 for SRCM103517, *p* = 0.0250 for SRCM103571, *p* = 0.0027 for SRCM103576, *p* = 0.0099 for SRCM103689, and *p* = 0.0000 for SRCM104011, analyzed by Kaplan–Meier analysis. (**B**) After *C. elegans* was exposed to bacteria for 24 h, the ability of the bacteria to attach to the nematode intestine was confirmed. Data are presented as the mean ± SD of three experiments. The error bars represent the standard deviation.

*C. elegans* has a relatively short lifespan of approximately 17 days on average and is thus considered as a model system suitable for aging research [48]. In many studies, *C. elegans* has been used to evaluate substances that prolong the life span [49]. To investigate whether the 5 selected *Bacillus* strains, showing higher expression levels of *pmk-1* than LGG, extend the lifespan of *C. elegans,* we performed a lifespan prolongation experiment. A total of ninety worms were exposed to candidate bacteria-treated plates, and the number of surviving worms was measured daily. *E. coli* OP50 was used as a control, which is a basic food of *C. elegans*. All 5 strains were shown to extend the lifespan of *C. elegans* compared with the control OP50 from 17 to 21 days (*p* < 0.05). *B. subtilis* SRCM 104011 extended the lifespan of the nematodes most significantly (*p* = 0.0000) (Figure 2A).

The life span is associated with stress, nutrition, and environmental signals and is regulated by conserved signaling pathways and transcription factors [50]. Metchnikoff hypothesized that consumption of milk and probiotics could increase the lifespan of the host, and continued research has shown that probiotics are positive for the longevity and health of the host [51,52]. In addition, probiotics extend the lifespan of *C. elegans* through various genetic pathways, such as p38 MAPK/SKN-1 or DAF-2/DAF-16, and through improvements in immune function [53,54,55]. Based on these findings, we speculate that the five strains evaluated in this study could increase the lifespan of nematodes contributing to the immunity of the host. Further research is needed to determine the more sophisticated mechanism of the isolated *Bacillus* actions for increasing the lifespan of nematodes.

### 3.4. Colonization of Bacteria on C. elegans Intestine

The human gastrointestinal tract is inhabited by a complex and dynamic community of approximately 500 to 1000 different microbial species. Among these, bacterial strains with identified beneficial properties include mainly *Bifidobacterium* and *Lactobacillus* species [56]. In general, to be considered as probiotics, these bacterial strains must survive the gastrointestinal tract [7] and adhere to the intestinal mucosa in their host [57,58]. To evaluate the probiotic potential of the isolated *Bacillus* species, we examined the intestinal adhesion of the five isolated strains in the nematodes after exposure for 24 h (Figure 2B). Two strains of *B. subtilis* (*B. subtilis* SRCM103571 and SRCM103576) showed adhesion of the intestine, but the other three strains were not adhered.

Factors for determining bacterial colonization to the host intestine are surface hydrophobicity, excretion of secretory enzymes and lipopeptides, and utilization of polysaccharide produced from the epithelial cells [59]. Many *B. subtilis* strains have an adhesive capability, but the degree is varied according to the strain and its physiological state [60]. Specific probiotics promote health by colonizing the host’s intestine, while some of them do transient probiotic colonization that are metabolically active in the intestine without replication to high numbers or displacing members of the native gut microbiota [61]. Here, the longevity effect of the *B. subtilis* strains on the *C. elegans* model suggested that the lifespan of the nematode model could be extended through other mechanisms in addition to colonizing the intestine of the *C. elegans* model.

### 3.5. Whole-Genome Sequencing and Pangenome Analysis

**Figure 3:**
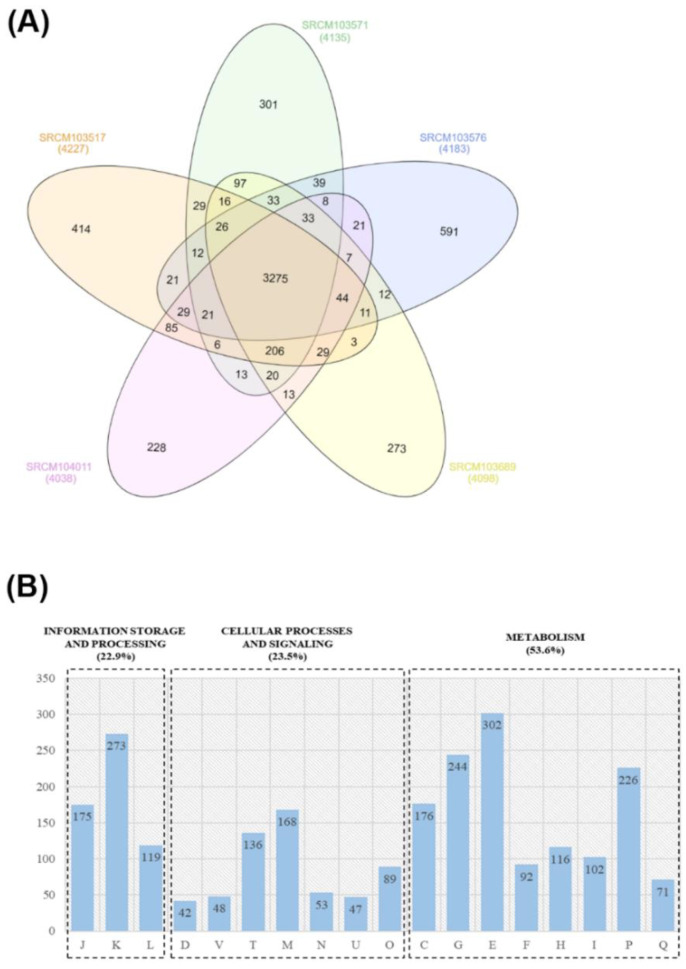
Venn diagram of pangenome analysis results using selected *B. subtilis* strains from Korean traditional fermented food. (**A**) Venn diagram of pangenome analysis results using 5 *B. subtilis* strains from Korea traditional fermented food. (**B**) Core genes of *B. subtilis* 5 strains from Korea using the COG database. J, Translation, ribosomal structure, and biogenesis; K, Transcription; L, Replication, recombination, and repair; D, Cell cycle control, cell division, and chromosome partitioning; V, Defense mechanisms; T, Signal transduction mechanisms; M, Cell wall/membrane/envelope biogenesis; N, Cell motility; O, Posttranslational modification, protein turnover, and chaperones; C, Energy production and conversion; G, Carbohydrate transport and metabolism; E, Amino acid transport and metabolism; F, Nucleotide transport and metabolism; H, Coenzyme transport and metabolism; I, Lipid transport and metabolism; P, Inorganic ion transport and metabolism; Q, Secondary metabolite biosynthesis, transport, and catabolism.

In order to provide genomic insights and unique features of the selected five strains, possibly associated with their host health-promoting effects, we first conducted whole-genome sequencing of the selected bacteria, SRCM103517, SRCM103571, SRCM103576, SRCM103689, and SRCM104011 (Supplementary Figure S2). General genome features of the *B. subtilis* strains are described in Supplementary Table S2. By comparing COG (Clusters of Orthologous Groups of proteins) data from the five isolated *B. subtilis* strains, 3275 core genes were clustered. The numbers of unique genes of the five strains confirmed the difference in genes for each strain. Of the five *Bacillus* strains, SRCM 103576 and SRCM104011 have a total of 591 and 228 unique genes that are the largest and the smallest number of unique genes among the five strains, respectively (Figure 3A). Analysis of the core gene functionality of the five strains confirmed that they are involved in information storage and processing (22.9%), cellular processes and signals (23.5%), and metabolism (53.6%) functions (Figure 3B).

**Figure 4:**
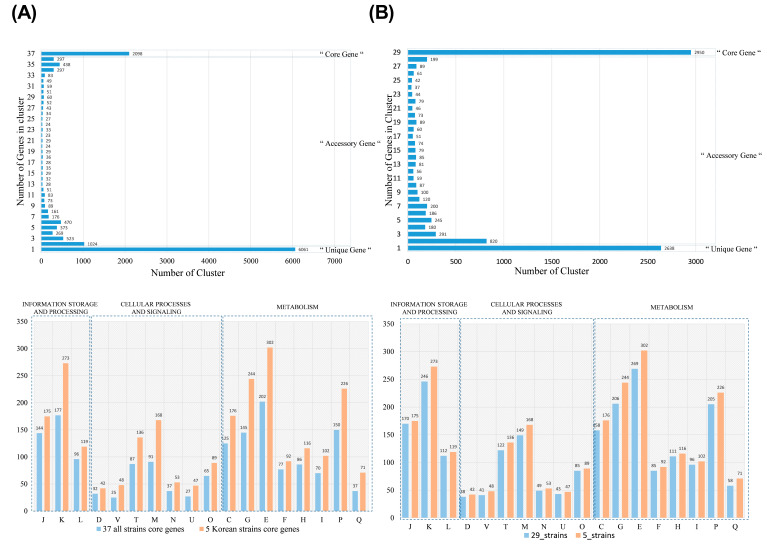
Pangenome analysis results using *B. subtilis* strains available with complete genome sequences in NCBI with selected *B. subtilis* strains from Korean traditional fermented food. (**A**) Upper: Pangenome analysis results using 32 *B. subtilis* strains available with complete genome sequences in NCBI and 5 strains from Korea in this study. Lower: Core genes of selected *B. subtilis* 5 strains from Korea and all 37 strains using the COG database. (**B**) Upper: Pangenome analysis results using 24 *B. subtilis* strains from Korea in another study available with complete genome sequences in NCBI and 5 strains from Korea in this study. Lower: Core genes of 5 *B. subtilis* strains from Korea in this study and all 29 strains from Korea in this and other studies using the COG database. J, Translation, ribosomal structure, and biogenesis; K, Transcription; L, Replication, recombination, and repair; D, Cell cycle control, cell division, and chromosome partitioning; V, Defense mechanisms; T, Signal transduction mechanisms; M, Cell wall/membrane/envelope biogenesis; N, Cell motility; O, Posttranslational modification, protein turnover, and chaperones; C, Energy production and conversion; G, Carbohydrate transport and metabolism; E, Amino acid transport and metabolism; F, Nucleotide transport and metabolism; H, Coenzyme transport and metabolism; I, Lipid transport and metabolism; P, Inorganic ion transport and metabolism; Q, Secondary metabolite biosynthesis, transport, and catabolism.

Pangenome analysis compares the entire gene set of all strains of a species, enabling assessment of the genomic diversity of entire repertoires of genes and identification of the core genomic elements [62]. To analyze the genomic differences among *B. subtilis* isolated from fermented products in East Asian countries, we next performed pangenome analysis using 61 genomes of different *B. subtilis* species, including 29, 3, and 24 *B. subtilis* strains isolated from China, Japan, and Korea, respectively, as well as the 5 strains isolated in this study. When compared the genomes of the 5 strains with the 32 *B. subtilis* strains isolated from China and Japan, a total of 2098 core genes were clustered, but less than the 3275 core genes clustered among the five strains in the above analysis, showing greater genetic differences by the origin of country. A group of 35 accessory genes were clustered, and 6061 unique genes were found (Figure 4A). According to the functional analysis of the core gene, the core gene was associated with metabolic, cellular processes and signals, information storage and processing functions. In particular, the five *B. subtilis* strains in this study had more functional genes than those from other East Asian *B. subtilis* strains (Figure 4A). Specifically, several genes, such as *deoA* (encoding pyrimidine-nucleoside phosphorylase), *haelllM_2* (encoding modification methylase HaeIII), *vgb* (encoding virginiamycin B lyase), and *vsr* (encoding very short patch repair protein), were present in only the currently isolated five strains (Table 1). Pyrimidine-nucleoside phosphorylase encoded by *deoA* catalyzes phosphoryolysis of the pyrimidine nucleosides with the formation of the pyrimidine base and ribose-1-phosphate. This enzyme participates in pyrimidine metabolism and has been studied for anticancer drug design [63]. Related to *deoA*, haeIIIM_2 is also involved in nucleotide metabolism as DNA methyltransferase, implementing that the isolated bacterial strains in this study would be associated with an altered nucleotide metabolism compared to the other *B. subtilis* strains. There is a study showing that the *vgb* gene, uniquely present in *B. subtilis* SRCM103517, SRCM103689, and SRCM104011 strains, increases intestinal tract colonization of mice and has antioxidant effects, thereby reducing liver toxicity in mice. To elucidate the molecular mechanisms of the isolated strains as probiotics and to enhance their applicability, further functional studies on these genes will be performed.

Next, we conducted a comparative analysis on the 5 *B. subtilis* strains in this study and previously reported 24 *B. subtilis* strains from Korean traditional foods to find unique features in the functionally selected five strains but not present in other strains originated from same country. As a result of the pangenome analysis of the 29 *B. subtilis* strains, it was confirmed that a total of 2950 core genes were clustered and that 27 accessory gene groups were clustered and had 2638 unique genes (Figure 4B). The core genes were involved in the functions on metabolic, cellular processes and signals, information storage and processing, and the strains in this study possessed more core genes compared to the other 24 strains previously isolated in Korea (Figure 4B). Average nucleotide identity (ANI) tree analysis revealed that the genomic characteristics of *B. subtilis* species would be clustered by the sources of isolation and that at least 4 strains in this study are correlated with the *B. subtilis* strains isolated from doenjang (Supplementary Figure S3). Importantly, we found unique genes present only in the five selected strains in this study, but not in other strains isolated from Korean fermented products as shown in Table 2. Among the five strains, SRCM103571 and SRCM103689 possessed a number of unique genes including *cscB, lacF_2*, *lacG*, *maa_1*, *nisB*, *nisC_2*, *sacA*, and *spaS*, which are involved in disaccharide permeases, sucrose-5-phosphate hydrolase, nisin biosynthesis, and lantibiotic subtilin synthesis. Disaccharide metabolism-associated proteins including fructooligosaccharide and galactooligosaccharide permeases have been reported to be essential for colonization, have effects on cell membrane fluidity, and be required for prebiotic utilization, providing the bacterial strains to attach and colonize the host intestine [64,65,66]. Additionally, peptides such as nisin and subtilin, encoded by *nisB*, *nisC*, and *spaS*, are bacteriocin effective against many Gram-positive organisms, functioning as probable probiotics with antimicrobial activities [67,68,69]. Moreover, *B. subtilis* SRCM103571 and SRCM103689 specifically possess genes encoding response regulators of aspartate phosphatase I and SaeR, which are involved in sporulation and adhesion to the host cells [70,71].

In the previous section, we demonstrated that the selected five *B. subtilis* strains induced high expression of *pmk-1* in the nematode host possibly through the p38-MAPK signaling pathway (Figure 1). The association of *B. subtilis* and its effects on the MAPK signaling has been suggested from many studies. For example, surfactin produced by *B. subtilis* upregulates the expression of MAPK signaling pathway and controls the secretion of proinflammatory cytokines, overall promoting wound healing and scar inhibition [72]. Sublancin, a glucosylated antimicrobial peptide produced in *B. subtilis*, modulates innate immunity via MAPK signaling pathway enhancing phagocytic activity of macrophages [73]. Moreover, pentapeptide competence and sporulation factor (CSF) of *B. subtilis* is a quorum sensing factor and controls competence and spore formation in the bacteria. A recent study reported that the factor is involved in the induction of cytoprotective heat shock protein in host intestinal cells via p38-MAPK signaling pathway [74]. In order to address the question of genetic differences between the five selected strains, functioning the induction of *pmk-1* on host, and other *B. subtilis* species isolated in Korea, we compared amino acid sequences of surfactin synthase subunits SrfAC and SrfAD in the total 29 *B. subtilis* strains. As shown in supplementary Figure S4, both protein sequences were highly conserved among the strains. The five strains in this study were shown to have more closely-related SrfAC than others. For SrfAD, the phylogenic analysis indicated that SRCM104011, SRCM103571, and SRCM103517 have slightly different amino acid sequences than other strains. These results implement that the five strains might have a distinct characteristic on the surfactin activity, probably associated with the host MAPK activity. To prove this hypothesis, further studies on the surfactin synthesis and its activity in these selected strains are required.

## 4. Conclusions

*B. subtilis* is a major fermenting microorganism in some Korean traditional fermented foods (cheonggukjang, doenjang, and gochujang), contributing to the flavor, texture, and nutritional value of the products, while it is also suggested to have potential health effects, such as anticancer, antioxidant, and fibrinolytic activity, on humans [75]. In this study, we showed that five strains of *B. subtilis* isolated from Korean traditional fermented foods possess acid and bile tolerance, antimicrobial activity, and functions in life extension and immune gene enhancement in the *C. elegans* in vivo model. In addition, as a result of pangenome analysis of the whole-genome sequencing data, the five strains possessed genetic differences from the bacterial strains isolated in East Asian countries and previously reported Korean strains. Their genetic features might be involved in the extension of lifespan in the nematode model via MAPK pathway, but more sophisticated evaluations are required. We expect that the newly isolated *B. subtilis* strains, present in natural fermented foods, can be applied as promising probiotics to be valuable as GRAS strains possibly enhancing human immune functionality and lifespan; thus, continuous research is needed to determine their detailed mechanism of action and applicability.

## Figures and Tables

**Table 1:** Newly isolated *Bacillus subtilis*-specific genes that are not present in Chinese and Japanese strains.

Gene	Gene Description	Strains
*deoA*	Thymidine phosphorylase	SRCM103571, SRCM103689
*haeIIIM_2*	Modification methylase HaeIII	SRCM103571, SRCM103689
*vgb*	Virginiamycin B lyase	SRCM103517, SRCM103689, SRCM104011
*vsr*	Very short patch repair protein	SRCM103571, SRCM103689
*group_6918*	Response regulator aspartate phosphatase I	SRCM103571, SRCM104011
*group_16459*	Chromosomal replication initiator protein DnaA	SRCM103517, SRCM103571
*group_16672*	Competence pheromone	SRCM103571, SRCM103689

**Table 2:** Newly isolated *B. subtilis*-specific genes that are not present in other strains isolated from Korean fermented products.

Gene	Gene Description	Strains
*comX*	Competence pheromone	SRCM103571, SRCM103689
*tagU_1*	Polyisoprenyl-teichoic acid--peptidoglycan teichoic acid transferase TagU	SRCM103517, SRCM103571, SRCM103689, SRCM104011
*cscB*	Sucrose permease	SRCM103571, SRCM103689
*lacF_2*	Lactose transport system permease protein LacF	SRCM103571, SRCM103689
*lacG*	Lactose transport system permease protein LacG	SRCM103571, SRCM103689
*maa_1*	Maltose O-acetyltransferase	SRCM103571, SRCM103689
*nisB*	Nisin biosynthesis protein NisB	SRCM103571, SRCM103689
*nisC_2*	Nisin biosynthesis protein NisC	SRCM103571, SRCM103689
*rapI*	Response regulator aspartate phosphatase I	SRCM103517, SRCM104011
*sacA*	Sucrose-6-phosphate hydrolase	SRCM103571, SRCM103689
*saeR*	Response regulator SaeR	SRCM103517, SRCM104011
*scrK_2*	Fructokinase	SRCM103517, SRCM104011
*spaS*	Lantibiotic subtilin	SRCM103571, SRCM103689

## Supplementary Materials

The following are available online at https://www.mdpi.com/2304-8158/9/12/1805/s1. Figure S1: Acid tolerance and bile tolerance; Figure S2: Circular maps of the selected *B. subtilis* strains; Figure S3: ANI tree analysis of *B. subtilis* 5 strains SRCM103517, SRCM103571, SRCM103576, SRCM103689, and SRCM104011 (in this study) and other 24 strains previously isolated in Korea; Figure S4: Phylogenetic analysis of amino acid sequences of surfactin synthase subunits SrfAC and SrfAD in the total 29 *B. subtilis* strains including 5 strains (in this study) and other 24 strains previously isolated in Korea. Table S1: Antimicrobial activity against five pathogenic bacteria *B. cereus* KCTC3624, *B. cereus* KCCM40935, *S. aureus* KCCM11335, *L. monocytogenes* KCCM43155, and *E. faecalis* KCCM11814; Table S2: General genome features of the *B. subtilis* SRCM103517, SRCM103571, SRCM103576, SRCM103689, and SRCM104011.
