# Supplementary material for: Comparative Genomic and Functional Evaluations of Bacillus subtilis Newly Isolated from Korean Traditional Fermented Foods
Source: Foods. 2020 Dec 4;9(12):1805. doi: 10.3390/foods9121805 (PMC7762004; doi:10.3390/foods9121805)
Supplement: Supplementary file 1 [file foods-09-01805-s001.pdf]

foods

MDPI

Article

Comparative genomic and functional evaluations of *Bacillus subtilis* newly isolated from Korean traditional fermented foods

Hye Jin Choi[1], Donghyun Shin[2], Minhye Shin[1], Bohyun Yun[3], Minkyoung Kang[4], Hee-Jong Yang[5], Do-Youn Jeong[5], Younghoon Kim[1]* and Sangnam Oh[4]*

[1]Department of Agricultural Biotechnology and Research Institute of Agriculture and Life Science, Seoul National University, Seoul 08826, Korea

[2]Department of Agricultural Convergence Technology, Jeonbuk National University, Jeonju 54896, Korea

[3]Department of Animal Science and Institute of Milk Genomics, Jeonbuk National University, Jeonju 54896, Korea

[4]Department of Functional Food and Biotechnology, Jeonju University, Jeonju 55069, Korea

[5]Microbial Institute for Fermentation Industry, Sunchang, Jeonbuk 56048, Korea

*Correspondence: ykeys2584@snu.ac.kr and osangnam@jj.ac.kr

Supplementary data

Figure S1. Acid tolerance and bile tolerance. Among the 30 candidate strains, 16 strains showed survival rates of 50% or higher at pH 2 (A), and 28 strains showed survival rates of 80% or higher at 0.3% bile acids (B). *B. subtilis* SRCM103612, the highest acid-resistant strain, showed 75.91% survival at pH 2, while *B. velezensis* SRCM103691, the highest bile acid-resistant strain, had 99.09% survival at 0.3% bile acid.

Figure S2. Circular maps of the selected *B. subtilis* strains. Circular maps of *B. subtilis* (A) SRCM103517, (B) SRCM103571, (C) SRCM103576, (D) SRCM103689, and (E) SRCM104011. The tables summarize the COG configuration. J, Translation, ribosomal structure and biogenesis; K, Transcription; L, Replication, recombination and repair; D, Cell cycle control, cell division, chromosome partitioning; V, Defense mechanisms; T, Signal transduction mechanisms; M, Cell wall/membrane/envelope biogenesis; N, Cell motility; O, Posttranslational modification, protein turnover, and chaperones; C, Energy production and conversion; G, Carbohydrate transport and metabolism; E, Amino acid transport and metabolism; F, Nucleotide transport and metabolism; H, Coenzyme transport and metabolism; I, Lipid transport and metabolism; P, Inorganic ion transport and metabolism; Q, Secondary metabolite biosynthesis, transport, and catabolism.

Figure S3. ANI tree analysis of *B. subtilis* 5 strains (in this study) and other 24 strains previously isolated in Korea.

Figure S4. Phylogenetic analysis of amino acid sequences of surfactin synthase subunits SrfAC (A) and SrfAD (B) in the total 29 *B. subtilis* strains including 5 strains (in this study) and other 24 strains previously isolated in Korea. The analysis was performed on the Phylogeny.fr platform (http://phylogeny.fr). Sequences were aligned with MUSCLE (v3.7), and ambiguous regions containing gaps or poorly aligned were removed with Gblocks (v0.91b) using default parameters. The tree was reconstructed using the maximul likelihood method implemented in the PhyML program (v3.0).

Table S1. Antimicrobial activity against five pathogenic bacteria *B. cereus* KCTC3624, *B. cereus* KCCM40935, *S. aureus* KCCM11335, *L. monocytogenes* KCCM43155 and *E. faecalis* KCCM11814.

Table S2. General genome features of the *B. subtilis* SRCM103517, SRCM103571, SRCM103576, SRCM103689, and SRCM104011.

Figure S1. Acid tolerance and bile tolerance. Among the 30 candidate strains, 16 strains showed survival rates of 50% or higher at pH 2 (A), and 28 strains showed survival rates of 80% or higher at 0.3% bile acids (B). *B. subtilis* SRCM103612, the highest acid-resistant strain, showed 75.91% survival at pH 2, while *B. velezensis* SRCM103691, the highest bile acid-resistant strain, had 99.09% survival at 0.3% bile acid.

(A)

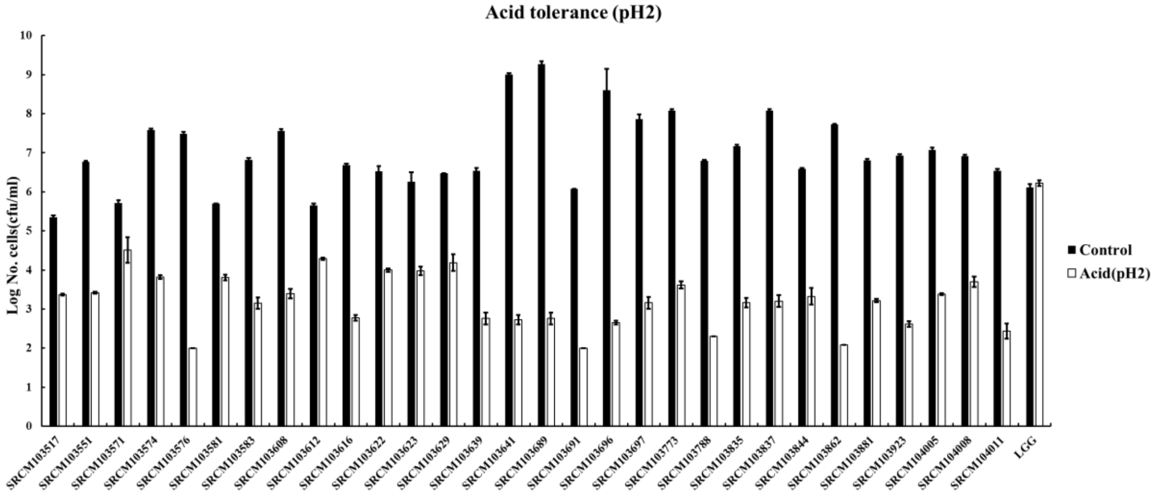

(B)

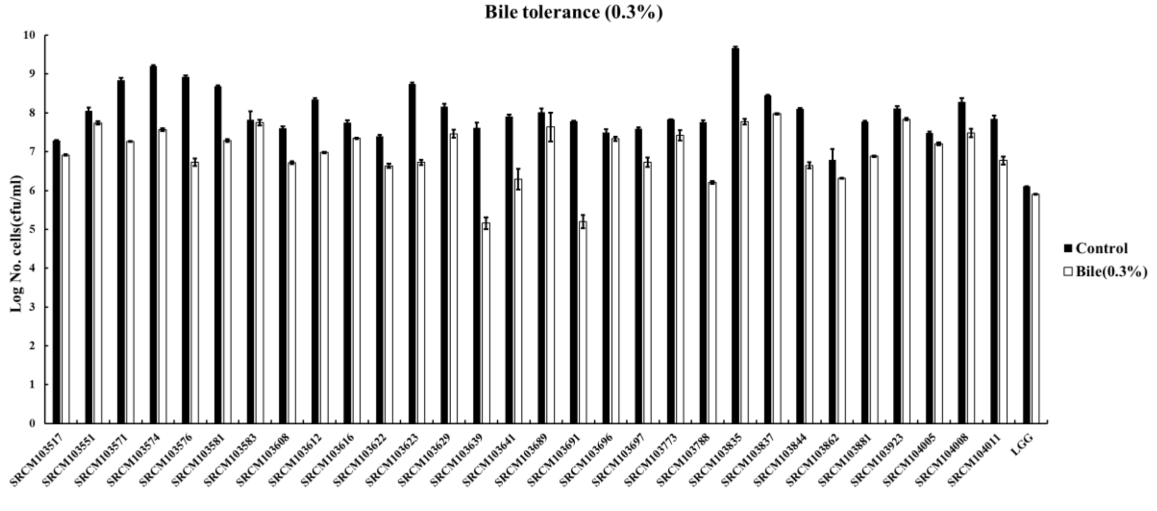

Figure S2. Circular maps of the selected *B. subtilis* strains. Circular maps of *B. subtilis* (A) SRCM103517, (B) SRCM103571, (C) SRCM103576, (D) SRCM103689, and (E) SRCM104011. The tables summarize the COG configuration. J, Translation, ribosomal structure and biogenesis; K, Transcription; L, Replication, recombination and repair; D, Cell cycle control, cell division, chromosome partitioning; V, Defense mechanisms; T, Signal transduction mechanisms; M, Cell wall/membrane/envelope biogenesis; N, Cell motility; O, Posttranslational modification, protein turnover, and chaperones; C, Energy production and conversion; G, Carbohydrate transport and metabolism; E, Amino acid transport and metabolism; F, Nucleotide transport and metabolism; H, Coenzyme transport and metabolism; I, Lipid transport and metabolism; P, Inorganic ion transport and metabolism; Q, Secondary metabolite biosynthesis, transport, and catabolism.

(A)

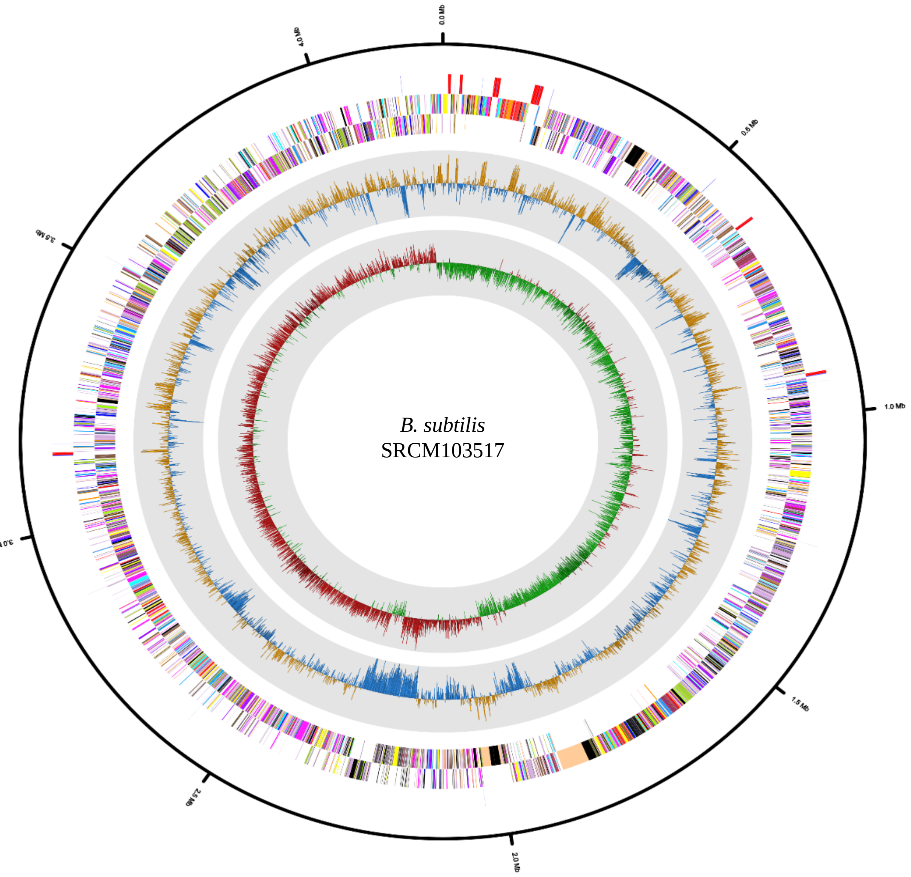

COG	Description	Number of Genes	%
J	Translation, ribosomal structure and biogenesis	163	4.33
K	Transcription	294	7.81
L	Replication, recombination and repair	149	3.96
D	Cell cycle control, cell division, chromosome partitioning	35	0.93
O	Posttranslational modification, protein turnover, chaperones	110	2.92
M	Cell wall/membrane/envelope biogenesis	225	5.98
N	Cell motility	43	1.14
P	Inorganic ion transport and metabolism	207	5.50
T	Signal transduction mechanisms	148	3.93
C	Energy production and conversion	184	4.89
G	Carbohydrate transport and metabolism	296	7.87
E	Amino acid transport and metabolism	313	8.32
F	Nucleotide transport and metabolism	88	2.34
H	Coenzyme transport and metabolism	107	2.84
I	Lipid transport and metabolism	99	2.63
Q	Secondary metabolites biosynthesis, transport and catabolism	62	1.65
R	General function prediction only	0	0.00
S	Function unknown	1240	32.95
Total		3763	100.00

(B)

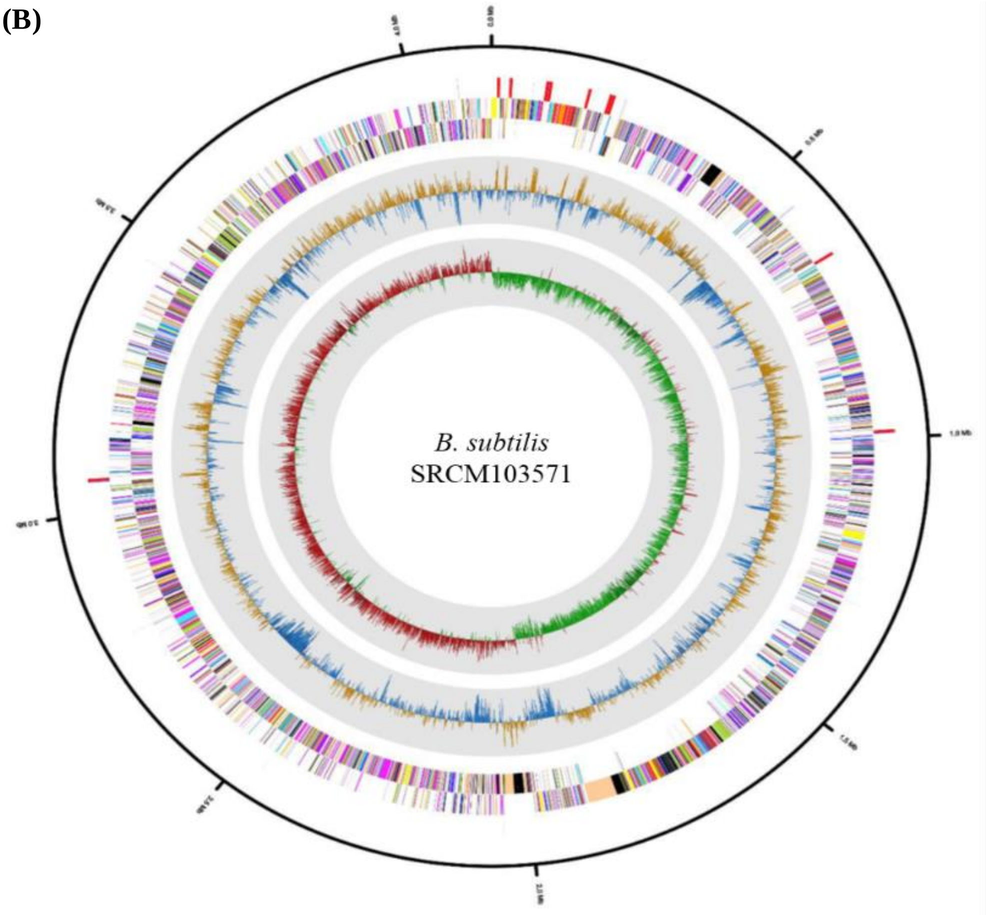

COG	Description	Number of Genes	%
J	Translation, ribosomal structure and biogenesis	162	4.34
K	Transcription	295	7.90
L	Replication, recombination and repair	143	3.83
D	Cell cycle control, cell division, chromosome partitioning	34	0.91
O	Posttranslational modification, protein turnover, chaperones	109	2.92
M	Cell wall/membrane/envelope biogenesis	220	5.89
N	Cell motility	42	1.13
P	Inorganic ion transport and metabolism	206	5.52
T	Signal transduction mechanisms	148	3.96
C	Energy production and conversion	178	4.77
G	Carbohydrate transport and metabolism	291	7.80
E	Amino acid transport and metabolism	305	8.17
F	Nucleotide transport and metabolism	83	2.22
H	Coenzyme transport and metabolism	108	2.89
I	Lipid transport and metabolism	101	2.71
Q	Secondary metabolites biosynthesis, transport and catabolism	62	1.66
R	General function prediction only	0	0.00
S	Function unknown	1246	33.38
Total		3733	100.00

(C)

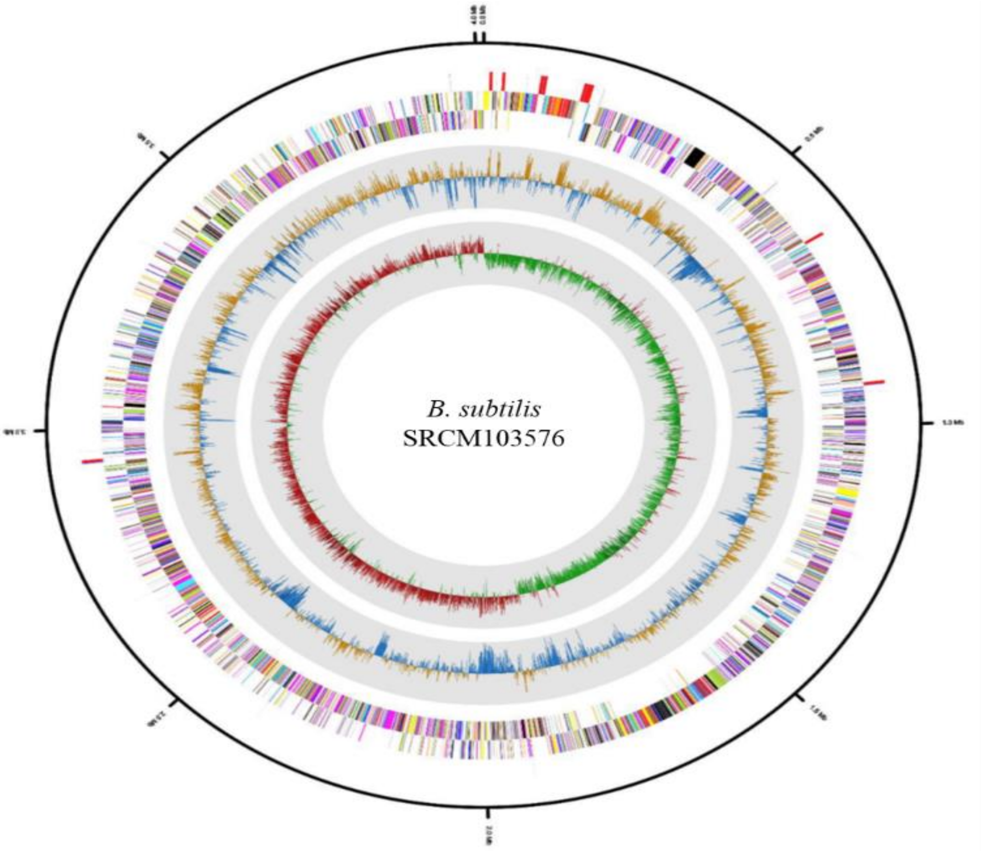

COG	Description	Number of Genes	%
J	Translation, ribosomal structure and biogenesis	166	4.45
K	Transcription	283	7.59
L	Replication, recombination and repair	192	5.15
D	Cell cycle control, cell division, chromosome partitioning	35	0.94
O	Posttranslational modification, protein turnover, chaperones	106	2.84
M	Cell wall/membrane/envelope biogenesis	229	6.14
N	Cell motility	44	1.18
P	Inorganic ion transport and metabolism	199	5.34
T	Signal transduction mechanisms	148	3.97
C	Energy production and conversion	171	4.59
G	Carbohydrate transport and metabolism	281	7.54
E	Amino acid transport and metabolism	299	8.02
F	Nucleotide transport and metabolism	82	2.20
H	Coenzyme transport and metabolism	107	2.87
I	Lipid transport and metabolism	93	2.49
Q	Secondary metabolites biosynthesis, transport and catabolism	51	1.37
R	General function prediction only	0	0.00
S	Function unknown	1242	33.32
Total		3728	100.00

(D)

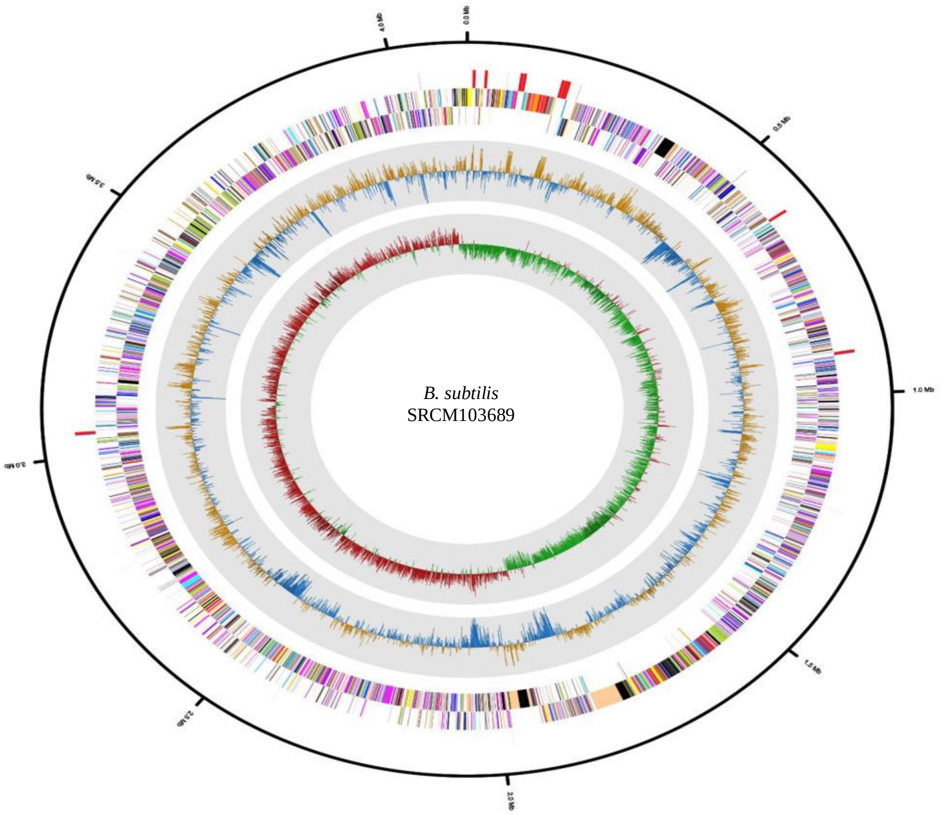

COG	Description	Number of Genes	%
J	Translation, ribosomal structure and biogenesis	164	4.43
K	Transcription	290	7.84
L	Replication, recombination and repair	134	3.62
D	Cell cycle control, cell division, chromosome partitioning	37	1.00
O	Posttranslational modification, protein turnover, chaperones	108	2.92
M	Cell wall/membrane/envelope biogenesis	222	6.00
N	Cell motility	44	1.19
P	Inorganic ion transport and metabolism	205	5.54
T	Signal transduction mechanisms	153	4.14
C	Energy production and conversion	174	4.70
G	Carbohydrate transport and metabolism	297	8.03
E	Amino acid transport and metabolism	303	8.19
F	Nucleotide transport and metabolism	88	2.38
H	Coenzyme transport and metabolism	106	2.87
I	Lipid transport and metabolism	100	2.70
Q	Secondary metabolites biosynthesis, transport and catabolism	60	1.62
R	General function prediction only	0	0.00
S	Function unknown	1214	32.82
Total		3699	100.00

Figure S3. ANI tree analysis of *B. subtilis* 5 strains SRCM103517, SRCM103571, SRCM103576, SRCM103689, and SRCM104011 (in this study) and other 24 strains previously isolated in Korea.

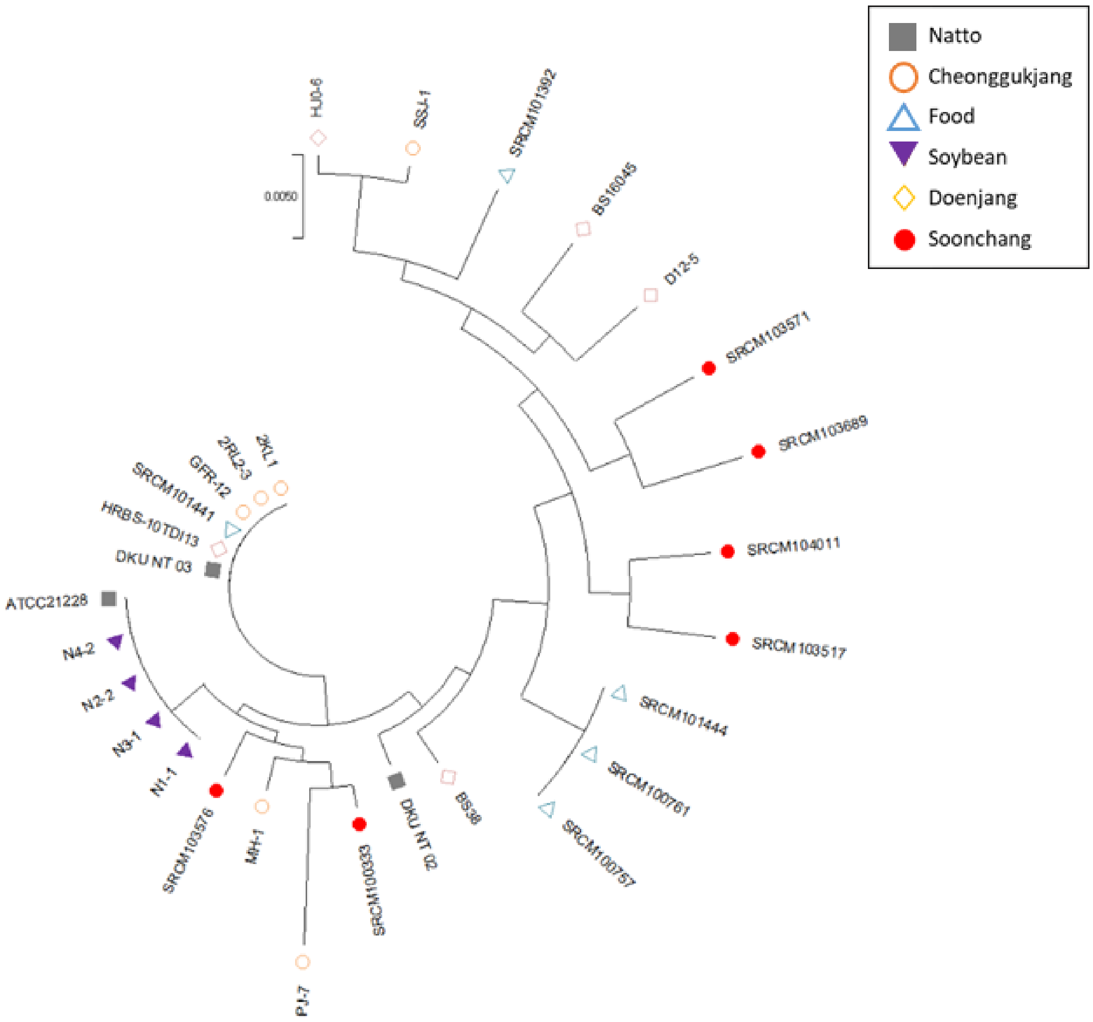

Figure S4. Phylogenetic analysis of amino acid sequences of surfactin synthase subunits SrfAC (A) and SrfAD (B) in the total 29 *B. subtilis* strains including 5 strains SRCM103517, SRCM103571, SRCM103576, SRCM103689, and SRCM104011 (in this study; indicated as stars) and other 24 strains previously isolated in Korea. The analysis was performed on the Phylogeny.fr platform (http://phylogeny.fr). Sequences were aligned with MUSCLE (v3.7), and ambiguous regions containing gaps or poorly aligned were removed with Gblocks (v0.91b) using default parameters. The tree was reconstructed using the maximul likelihood method implemented in the PhyML program (v3.0).

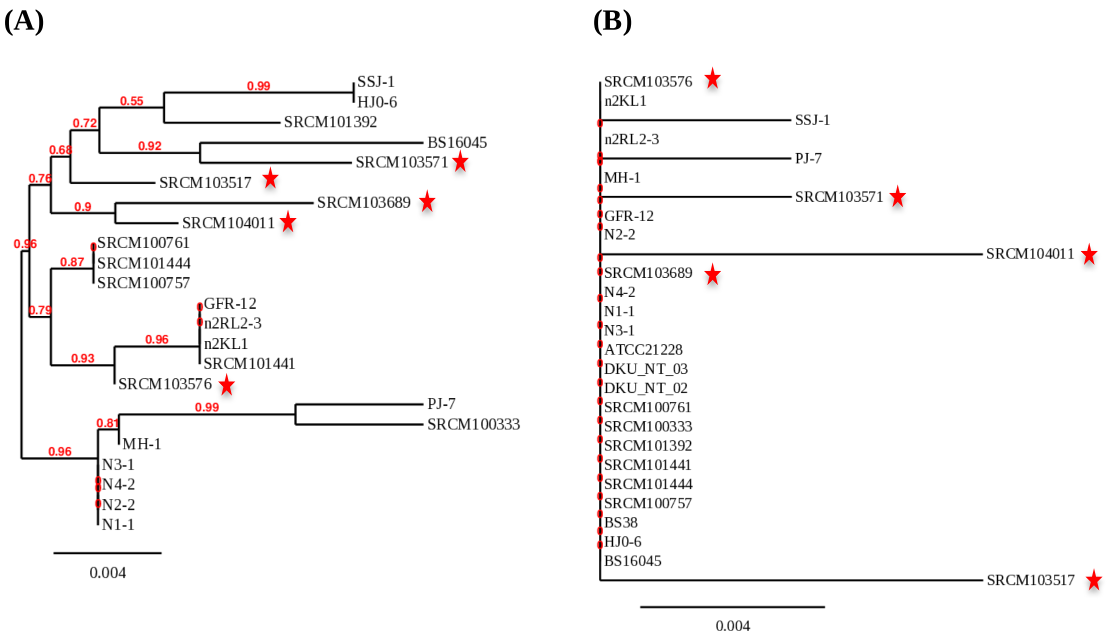

Table S1. Antimicrobial activity against five pathogenic bacteria *B. cereus* KCTC3624, *B. cereus* KCCM40935, *S. aureus* KCCM11335, *L. monocytogenes* KCCM43155 and *E. faecalis* KCCM11814. bacteria antimicrobial activity was evaluated to the size of the inhibition zone. (ND, no inhibition zone).

Strain No.	Identification	Anti-microbial effect (cm)				
		B. cereus KCTC 3624	*B. cereus* KCCM 40935	*S. aureus* KCCM 11335	*L. monocytogenes* KCCM 43155	*E. faecalis* KCCM 11814
SRCM103517	*B. subtilis*	1.6	1.5	1.3	1.6	1.4
SRCM103551	*B. subtilis*	1.4	1.3	ND	ND	1.2
SRCM103571	*B. subtilis*	2.0	1.7	1.5	2.0	1.2
SRCM103574	*B. licheniformis*	1.5	1.0	1.2	1.3	1.1
SRCM103576	*B. subtilis*	ND	ND	0.9	1.5	1.3
SRCM103581	*B. subtilis*	1.7	1.2	1.1	2.2	1.2
SRCM103583	*B. licheniformis*	1.5	1.1	ND	1.0	ND
SRCM103608	*B. licheniformis*	ND	ND	1.0	1.5	1.2
SRCM103612	*B. subtilis*	1.5	1.2	1.0	ND	1.2
SRCM103616	*B. velezensis*	1.2	ND	1.1	1.2	ND
SRCM103622	*B. subtilis*	1.8	1.5	1.5	1.8	1.5
SRCM103623	*B. subtilis*	1.5	1.0	1.2	2.0	1.3
SRCM103629	*B. subtilis*	1.7	1.0	1.3	2.0	1.3
SRCM103639	*B. velezensis*	1.1	1.4	1	ND	ND
SRCM103641	*B. subtilis*	1.8	1.3	1.1	1.9	ND
SRCM103689	*B. subtilis*	1.4	1.5	1.2	ND	ND
SRCM103691	*B. velezensis*	ND	ND	1	1.2	ND
SRCM103696	*B. subtilis*	1.5	1.6	1.4	ND	1.2
SRCM103697	*B. subtilis*	1.0	1.1	1.2	ND	ND
SRCM103773	*B. subtilis*	1.2	1.2	1.1	1.6	ND
SRCM103788	*B. velezensis*	ND	ND	ND	ND	ND
SRCM103835	*B. subtilis*	1.1	1.3	1.1	1.6	ND
SRCM103837	*B. subtilis*	1.5	1.6	1.1	2.0	1.4
SRCM103844	*B. licheniformis*	1.1	1.2	ND	1.2	ND
SRCM103862	*B. subtilis*	1.5	1.6	1.3	1.8	ND
SRCM103881	*B. subtilis*	1.3	1.5	1.4	2.0	1.4
SRCM103923	*B. subtilis*	1.5	1.5	1.6	ND	1.9
SRCM104005	*B. subtilis*	1.3	1.5	1	2.0	1.3
SRCM104008	*B. subtilis*	1.4	1.5	1.6	1.5	1.5
SRCM104011	*B. subtilis*	1.5	1.5	1.6	1.5	1.6
SRCM103886	*B. subtilis*	1.0	ND	1.1	ND	1.0
SRCM103914	*B. licheniformis*	1.2	1.4	0.9	1.2	1.0
SRCM103971	*B. subtilis*	1.0	1.1	1.4	ND	1.3
SRCM103529	*B. licheniformis*	1.3	1.2	ND	1.2	ND
SRCM103637	*B. subtilis*	1.7	1.5	0.8	1.8	ND

Table S2. General genome features of the *B. subtilis* SRCM103517, SRCM103571, SRCM103576, SRCM103689, and SRCM104011.

	SRCM103517	SRCM103571	SRCM103576	SRCM103689	SRCM104011
Genome size (bp)	4,215,632	4,133,685	4,012,178	4,123,786	4,105,116
GC contents (%)	43.57	43.81	43.5	43.96	43.79
Number of ORFs	4,225	4,135	4,187	4,095	4,037
Number of tRNA genes	86	86	86	87	86
Number of rRNA genes	30	30	30	30	27
